# Supplementary material for: The impact of environmental education at Chinese Universities on college students’ environmental attitudes
Source: PLoS One. 2024 Feb 29;19(2):e0299231. doi: 10.1371/journal.pone.0299231 (PMC10903793; doi:10.1371/journal.pone.0299231)
Supplement: Attachment — Submitted filename: Response to Reviewers.docx [file pone.0299231.s002.docx]

**Rebuttal Letter for [PONE-D-23-29584]**

Dear Bo Pu, Ph.D.,

We appreciate the thorough review conducted by the academic editors and reviewers on our manuscript titled "The Impact Environmental Education at Chinese Universities on College Students' Environmental Attitudes" submitted to PLOS ONE. We have carefully considered each comment and made the necessary revisions to address the concerns raised. Below, we respond to each point raised by the academic editors and reviewers:

Academic Editors' Comments:

1. File Naming and Style Requirements:

We have revised the file naming according to PLOS ONE's style requirements.

2. Raw Data Deposit:

We acknowledge the importance of data accessibility and have deposited our raw data in a repository to enhance visibility, appreciation, and citation. The data is stored in a GitHub repository, accessed at:

<https://github.com/LeeYibo/Dissertation-data-on-ecological-attitudes>

3. Title Correction:

We have changed the title of the article to "The Impact of Environmental Education at Chinese Universities on College Students' Environmental Attitudes" and ensured that the title is consistent between the online submission form and the manuscript.

4. Reference List:

The reference list has been thoroughly reviewed and corrected. No retracted papers in the reference list.

Reviewer #1's Comments:

1. Description of Variables and Scales:

We have provided a comprehensive description of the variables and scales used for measurement, including their names and psychometric properties in our context.

2. Title Revision:

The title has been revised (Academic Editors' Comments: 3.)

3. Year of Study:

The year of study has been added to the manuscript for clarity. ( 3.2. data sources and basic information about the sample)

4. Implications in Conclusion:

We have expanded the conclusion section to include more explicit implications of the study for our context.

Reviewer #2's Comments:

1. Reporting Means and Standard Deviations:

Means and standard deviations have been reported together as per PLOS ONE style guidelines. (Table 4. &Table 5.)

2. Table 6 Modification:

The second column of Table 6, containing redundant information, has been removed for clarity. (remove the second column: Pearson's correlation)

3. Separation of Discussion and Conclusion:

We have separated the discussion and conclusion sections to enhance the structure of the manuscript.

4. Text Review and Correction:

We have thoroughly reviewed the manuscript and corrected any omissions and errors. The corrections are indicated in Revised Manuscript with Track Changes through the revision mode (Including the correction of line 385 regarding statistical significance, revised line 388.)

5. Hypothesis Presentation:

We have revised the presentation of hypotheses to state them in a more reader-friendly manner.

We believe that these revisions substantially improve the quality and compliance of our manuscript with PLOS ONE guidelines. We appreciate the time and effort invested by the academic editors and reviewers in evaluating our work.

Thank you for your consideration.

Sincerely,

Yibo Li

PONE-D-23-29584
